# Which questionnaires should we use to evaluate quality of life in patients with chronic graft-vs-host disease?

**DOI:** 10.3325/cmj.2016.57.6

**Published:** 2016-02

**Authors:** Zinaida Perić, Lana Desnica, Nadira Duraković, Alen Ostojić, Dražen Pulanić, Ranka Serventi-Seiwerth, Ema Prenc, Grzegorz Basak, Radovan Vrhovac, Steven Z Pavletic, Damir Nemet

**Affiliations:** 1 Department of Internal Medicine, University of Zagreb, School of Medicine, Zagreb, Croatia; 2 Department of Internal Medicine, Division of Hematology, University Hospital Centre Zagreb, Zagreb, Croatia; 3 Faculty of Medicine Osijek, J.J. Strossmayer University of Osijek, Osijek, Croatia; 4 Croatian Cooperative Group for Hematologic Diseases, Zagreb, Croatia; 5 Department of Hematology, Oncology and Internal Diseases, Medical University of Warsaw, Warsaw, Poland; 6 National Cancer Institute, Bethesda, MD, USA

## Abstract

**Aim:**

To investigate the ability of two standard quality of life (QOL) questionnaires – The Short Form (36-item) Health Survey (SF-36) and The European Organisation for Research and Treatment of Cancer Quality of Life Questionnaire-Core 30 (EORTC QLQ C30) to evaluate QOL in patients with chronic graft-vs-host disease (cGVHD) graded according to National Institutes of Health (NIH) consensus criteria.

**Methods:**

In this cross-sectional study, QOL was assessed in patients who underwent allogeneic stem cell transplantation (allo-SCT) at the University Hospital Centre Zagreb and were alive and in complete remission for more than one year after allo-SCT.

**Results:**

The study included 58 patients, 38 patients with cGVHD and 20 controls, patients without cGVHD. Patients with cGVHD scored according to the NIH criteria had significantly lower scores of global health status and lower QOL on all SF-36 subscales and most of QLQ C30 functional subscales (*P* < 0.050 for all comparisons). Furthermore, patients with active cGVHD had significantly lower QOL scores than patients with inactive cGVHD, and this difference was most evident in physical functioning subscale of SF-36 (*P* = 0.0007) and social functioning subscale of QLQ C30 (*P* = 0.009).

**Conclusion:**

cGVHD scored according to the NIH criteria is correlated with patient-reported QOL, particularly in the physical domains as detected by SF-36. QLQ C30 questionnaire adds more information on social functioning and should be used as a valuable tool in the evaluation of social domains in cGVHD patients.

Although it is potentially lifesaving for a variety of hematological malignant and non-malignant disorders, allogeneic stem cell transplantation (allo-SCT) carries a significant risk of acute and late post-transplant complications. Improvements in transplantation techniques and supportive care have resulted in a reduction of early transplant-related mortality (1,2). However, the burden of late complications remains high, and two thirds of long-term allo-SCT survivors experience at least one chronic health condition (3). These complications occur due to treatment exposures before and during allo-SCT, cause substantial mortality, and severely impair patients’ functional status and quality of life (QOL). This is why today the aim of the treatment is not just to cure the primary hematological disease, but to facilitate the recovery of the physical and emotional functioning and improve QOL and social reintegration in family and work environment.

Health-related QOL is now considered to be one of the relevant treatment outcomes because it provides a broader understanding of the patient’s status beyond simple disease-free survival. It is a multi-dimensional construct comprised of several related domains including physical, emotional, social, and role functioning, as well as a person's overall evaluation of his or her well-being and ability to function (4,5). Better understanding of QOL in long-term survivors is necessary to provide adapted pre-transplant counseling and recommendations for post-transplant follow-up.

With a cumulative incidence of 40%-70% and significant mortality, chronic GVHD (cGVHD) represents the most important late complication following allo-SCT (6,7). Moreover, it seems that the incidence of cGVHD in the recent years has been increasing, probably due to the fact that much older patients undergo allo-SCT, as well as due to the increased use of peripheral blood stem cell grafts and matched unrelated donors, all known risk factors for cGVHD (8). In a series of publications originating from 2005 National Institutes of Health (NIH) Consensus Conference, investigators proposed means to standardize diagnosis, scoring, histopathology, biomarkers, response assessment, and research in cGVHD (9-14). These criteria were developed to advance clinical trials and consequently improve the management of cGVHD and long-term survivorship after allo-SCT.

As one of the important treatment outcomes, QOL is increasingly being subjected to the same degree of rigorous study as other significant allo-SCT outcomes. Most of the studies so far have reported a negative, significant association between cGVHD and QOL after allo-SCT (15-20). However, some of the studies have found no association (21-25), and this relationship still needs to be elucidated. The awareness of the relationship between QOL and cGVHD is necessary to further facilitate the prevention and treatment of cGVHD.

The aim of this study was to examine the effect of cGVHD on QOL in our cohort of long-term allo-SCT survivors with the use of two standard QOL questionnaires; The Short Form (36-item) Health Survey (SF-36) and The EORTC Quality of Life Questionnaire (QLQ C30). Furthermore, we assessed QOL according to cGVHD severity and activity defined by the NIH consensus criteria.

## Patients and methods

### Study design

This cross-sectional study was part of a larger project titled “Clinical and Biological Factors Determining Severity and Activity of Chronic GVHD after Allogeneic Hematopoietic Stem Cell Transplantation” funded by the Unity Through Knowledge Fund. This project was conducted at the University Hospital Centre Zagreb and received ethical approval from the same institution. The project included all patients referred to hematologist for the evaluation of cGVHD independently of age or underlying diagnosis. Between July 2013 and October 2015, total of 76 patients, 47 cGVHD patients and 29 controls were included in the project.

The inclusion criteria for this study were patients in the project who were alive and in complete remission for more than one year after allo-SCT. Children were excluded from this study. From 60 patients who met the criteria for the study, two refused to participate, one due to cGVHD-related sight problems and another due to personal reasons. Fifty-eight patients who accepted to participate signed an informed consent for the study.

### Chronic GVHD evaluation

cGVHD was evaluated and scored according to the NIH consensus criteria (14). No-cGVHD cohort of patients were patients who had never had signs of cGVHD, irrespectively of their acute GVHD status. If they had standard or late-acute cGVHD, it had to be resolved and they did not receive any immunosuppression at the time of evaluation. For patients with established cGVHD diagnosis, additional data regarding the severity and activity of disease were collected. Disease severity was defined according to the global NIH scoring: mild cGVHD involved only 1 or 2 organs (except lungs, maximum score 1 in all affected organs). Moderate cGVHD involved at least 1 organ with clinically significant but not major disability (maximum score 2) or 3 or more organs with no clinically significant functional impairment (maximum score 1 in all affected organs). Lung score 1 was classified as moderate. Severe cGVHD indicated major impairment caused by cGVHD (score 3 in any organ). Lung scores of 2 or 3 were classified as severe. Organs scored included the skin, eyes, mouth, gastrointestinal tract, liver, lungs, and joint/fascia. The genital area was scored only in women (14). In order to establish disease activity we noted a) Clinicians’ impression of activity previously defined by Grkovic et al (26) as inactive off systemic therapy or topical immunosuppression, inactive on systemic therapy or topical immunosuppression, active irrespective of the level of current therapy, or highly active irrespective of the level of current therapy, and b) intensity of immunosuppression at the time of evaluation defined as: none, mild = single agent prednisone <0.5 mg/kg/d; moderate = prednisone ≥0.5 mg/kg/d and/or any single agent/modality; high = 2 or more agents/modalities ± prednisone ≥0.5 mg/kg/d (27).

### Quality of life instruments

Patients received two questionnaires at the time of project enrollment: SF-36 and EORTC QLQ-C30. SF-36 is a 36-item self-report questionnaire that assesses patient-reported health and functioning. The instrument examines the following domains of QOL: physical functioning, role limitations due to physical health, role limitations due to emotional problems, energy/fatigue, emotional well-being, social functioning, pain, and general health (28,29). SF-36 was translated and validated in Croatian at the School of Public Health “Andrija Štampar” for the purposes of the health care analysis project and later used in many different studies in Croatia (30-32).

The EORTC QLQ-C30 (33) is a cross-culturally validated questionnaire of 30 questions, which make 5 multi-item functional scales (physical, role, emotional, cognitive, and social functioning), and a combined global health status/QOL scale. Higher scores on these scales indicate better functioning (33). Three symptom scales measure fatigue, pain, nausea, and vomiting, while 6 single items assess symptoms commonly reported by cancer patients (dyspnea, sleep disturbances, appetite loss, diarrhea, constipation, and financial impact). Higher scores on the symptom scales and single items represent greater higher impairments. QLQ-C30 version translated and validated in Croatian was obtained for this research from the EORTC QOL group headquartered in Belgium.

### Statistical methods

All analyses were performed using the R package (34). Data are presented as median and range. Comparisons of global QOL, physical, role, emotional, social functioning, and symptom scales between patients with and without cGVHD and between patients with moderate and severe cGVHD, were evaluated by a non-parametric Mann-Whitney U test. Comparisons between groups with regard to activity of cGVHD and level of immunosuppression were done with Kruskal-Wallis test. *P* value <0.050 was considered significant.

## Results

### Study population characteristics

Patients’ and graft characteristics are presented in Table 1. The study included 30 male and 28 female patients with a median age of 43 years (range 18-71). Most of the patients (69%) underwent transplantation due to myeloid malignancies, 22% due to lymphoid malignancies, and 9% due to aplastic anemia. The majority of patients (60%) underwent transplantation after myeloablative conditioning, while 40% received reduced-intensity conditioning prior to allo-SCT. In 62% of patients the donor was related to the patients and in 38% of patients the donor was unrelated. Finally, the majority of the patients received peripheral blood stem cells (57%), while the rest (43%) received the bone marrow. The median follow-up after allo-SCT was 659 days (range 361-7853). Baseline clinical characteristics were similar in patients with and without cGVHD (Table 1).

**Table 1:** Demographic characteristics of the study population, N (%)

Characteristic	Patients without cGVHD* (n = 20)	Patients with cGVHD (n = 38)	All patients (n = 58)	*P* (Mann-Whitney)
Patient age median (range)	42 (18-71)	44 (21-56)	43 (18-71)	0.312
Sex				
male	10 (50)	19 (50)	30 (52)	
female	10 (50)	19 (50)	28 (48)	1.00
Diagnosis				
myeloid malignancies	14 (70)	26 (68)	40 (69)	
lymphoid malignancies	5 (25)	8 (21)	13 (22)	
aplastic anemia	1 (5)	4 (11)	5 (9)	0.832
Stem cell source				
bone marrow	9 (45)	16 (42)	25 (43)	
peripheral blood stem cells	11 (55)	22 (58)	33 (57)	1.00
Donor type				
matched related donor	11 (55)	25 (66)	36 (62)	
matched unrelated donor	9 (45)	13 (44)	22 (38)	0.583
Conditioning regimen				
myeloablative	12 (60)	23 (60)	35 (60)	
reduced-intensity	8 (40)	15 (40)	23 (40)	1.00
Days from transplant to enrolment, median (range)	713 (361-5518)	606(367-7853)	659 (361-7853)	0.472
*cGVHD – chronic graft vs-host disease.				

### Chronic GVHD characteristics

cGVHD characteristics are shown in Table 2. 20 patients did not meet the NIH criteria for cGVHD diagnosis. In the no cGVHD group, 60% of patients previously had had acute GVHD but it completely resolved at the time of evaluation and they did not receive any immunosuppressive therapy.

**Table 2:** Characteristics of chronic graft-vs-host disease (cGVHD) patients

Characteristic	N (%)
cGVHD	
mild	1 (2)
moderate	17 (45)
severe	20 (53)
cGVHD days after transplantation median (range)	298 (103-3886)
cGVHD onset	
de novo	8 (21)
progressive	12 (32)
quiescent	18 (47)
cGVHD classification time of evaluation	
classic	35 (92)
overlap	3 (8)
cGVHD organs involved median (range)	2 (1-6)
cGVHD lines of immunosuppression median (range)	2 (1-4)
Clinician’s impression of cGVHD activity	
inactive of systemic or topical immunosuppression	13 (34)
inactive on systemic or topical immunosuppression	5 (13)
active irrespective of immunosuppression	17 (45)
highly active irrespective of immunosuppression	3 (8)
Intensity of immunosuppression at time of evaluation	
none	20 (53)
moderate	11 (29)
high	7 (18)

cGVHD was diagnosed in 38 patients; mild in 1, moderate in 17, and severe in 20 patients, at a median of 298 days after HSCT (range 103-3886). 79% of cGVHD patients previously had acute GVHD. cGVHD onset was quiescent in the majority of patients (47%), progressive in 32%, and it occurred de novo in 21% of the patients. At the time of the evaluation, cGVHD was classified as classic in 92% of the patients, and in 8% of the patients it overlapped with acute GVHD. The median number of cGVHD organs involved was 2 (range 1-6), and patients received a median of 2 (range 1-4) previous lines of immunosuppressive treatment. According to the clinical impression, 53% of patients had active or even highly active cGVHD irrespectively of the immunosuppressive treatment, while 34% had inactive cGVHD and were off immunosuppressive therapy. The remaining 13% of the patients had inactive cGVHD but still received immunosuppressive treatment. The majority of patients (53%) received no immunosuppressive treatment at the time of enrolment, 29% were treated with moderate immunosuppression, while 18% of patients received high-dose immunosuppressive therapy.

### Quality of life

**Figure 1:**
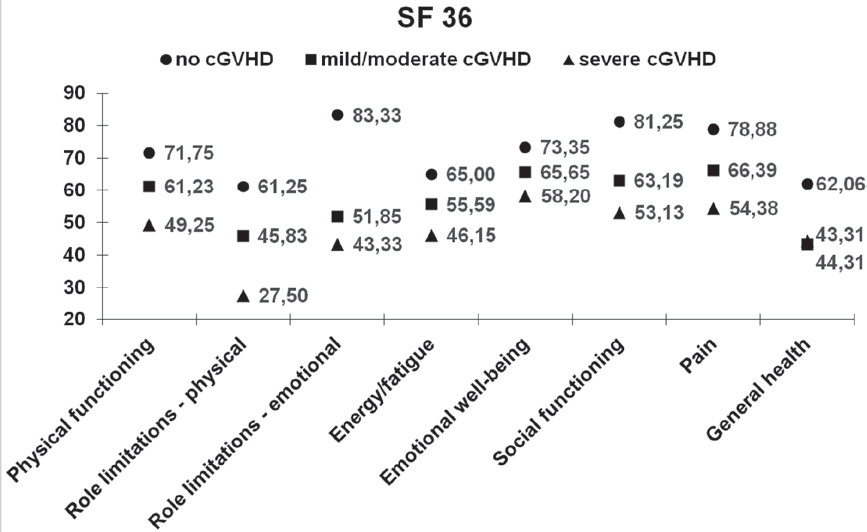
Mean scores in The Short Form (36) Health Survey (SF-36) questionnaire subscales in controls and in patients with mild/moderate and severe chronic graft-vs-host disease (cGVHD).

**Figure 2:**
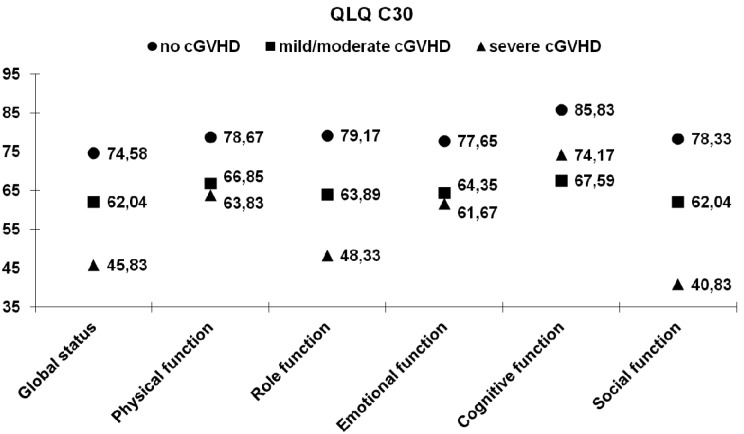
Mean scores in The EORTC Quality of Life Questionnaire C30 (QLQ C30) questionnaire functional subscales in controls and in patients with mild/moderate and severe chronic graft-vs-host disease (cGVHD).

In the analysis of QOL of all patients, we found that female patients, patients older than 50 years, and patients who fulfilled the questionnaires less than two years after allo-SCT had lower QOL scores than men, younger patients, or patients with longer follow up, although this difference did not reach statistical significance (data not shown). On the other hand, compared to patients without cGVHD, patients with cGVHD had significantly lower mean scores on all QOL subscales of the SF 36 questionnaire. Namely, cGVHD patients had significantly lower scores on physical functioning subscale, more role limitations due to physical and emotional functioning, less energy, worse emotional well-being and social functioning, more pain, and worse general health (*P* < 0.050 for all comparisons) (Table 3). cGVHD patients also had significantly lower QOL scores on QLQ C30 global health status and on most functional subscales (physical, role, emotional, and social) (*P* < 0.050 for all subscales). The difference was not significant only for cognitive functioning (Table 4). Moreover, on QLQ C30 symptom scales, cGVHD patients reported significantly more pain, dyspnea, and sleeping disorders (*P* = 0.010, *P* = 0.043, and *P* = 0.043, respectively) (Table 4). There was no difference in the scores for other symptoms: fatigue, nausea and vomiting, constipation, diarrhea, appetite loss, and financial difficulties. When we evaluated the severity of the cGVHD, patients with severe cGVHD had lower QOL scores than patients with mild/moderate cGVHD on both SF-36 (Figure 1) and QLQ C30 questionnaires (Figure 2). This difference was greatest in physical functioning and role limitations due to physical health on SF-36, as well as in global status and social functioning scores on QLQ C30, but it did not reach statistical difference. According to clinician's impression, we further divided the patients with cGVHD into those with active disease (active or highly active irrespectively of immunosuppressive treatment) and inactive (irrespectively of immunosuppressive therapy). Patients with active cGVHD had consistently lower QOL SF-36 scores, ie, significantly lower physical functioning, role limitations due to physical health, and social functioning, as well as less energy/more fatigue (*P* = 0.0007, *P* = 0.026, *P* = 0.017, and *P* = 0.014, respectively) (Table 5). Patients with active cGVHD also had significantly lower QLQ C30 scores, ie, worse physical and role functioning, as well as global health, and social functioning (*P* = 0.015, *P* = 0.011, *P* = 0.029, and *P* = 0.009, respectively (Table 6). Finally we examined QOL in patients with cGVHD according to the level of immunosuppressive treatment at the time of evaluation. Patients with high and moderate intensity of immunosuppression had significantly lower scores on physical functioning subscale (*P* = 0.044) and role limitations due to physical health (*P* = 0.031) of SF-36 (Table 5) and on general health subscale (*P* = 0.039), as well as more pain on the symptom scales (*P* = 0.033) of QLQ C30 (Table 6).

**Table 3:** Mean scores in The Short Form (36) Health Survey (SF-36) questionnaire subscales in patients with and without chronic graft-vs-host disease (cGVHD)

SF-36 mean (standard deviation)	Patients without cGVHD (n = 20)	Patients with cGVHD (n = 38)	*P* (Mann-Whitney)
Physical functioning	72.86 (24.06)	53.84 (26.27)	**0.007**
Role limitations due to physical health	63.10 (42.29)	34.46 (40.97)	**0.025**
Role limitations due to emotional problems	84.13 (32.69)	45.94 (44.69)	**0.004**
Energy/Fatigue	66.19 (18.30)	49.39 (19.04)	**0.005**
Emotional well-being	73.86 (13.71)	61.00 (19.23)	**0.017**
Social functioning	82.14 (19.19)	56.76 (26.29)	**0.0004**
Pain	78.93 (22.84)	59.53 (28.30)	**0.008**
General health	62.20 (21.42)	43.26 (19.62)	**0.018**

**Table 4:** Mean scores in The EORTC Quality of Life Questionnaire C30 (QLQ C30) questionnaire subscales in patients with and without chronic graft-vs-host disease (cGVHD)

QLQ C30 mean (standard deviation)	Patients without cGVHD (n = 20)	Patients with cGVHD (n = 38)	*P* (Mann-Whitney)
Global health status	75.00 (21.25)	52.70 (25.16)	**0.001**
Functional scales			
Physical functioning	79.05 (20.36)	64.68 (24.09)	**0.022**
Role functioning	80.16 (29.16)	54.50 (33.94)	**0.005**
Emotional functioning	78.32 (21.79)	62.16 (25.05)	**0.017**
Cognitive functioning	85.71 (12.12)	70.72 (28.17)	0.084
Social functioning	79.37 (18.93)	49.55 (32.98)	**0.001**
Symptom scale			
Fatigue	28.78 (20.53)	42.26 (26.92)	0.063
Nausea and vomiting	4.76 (10.73)	10.36 (19.39)	0.312
Pain	17.46 (25.54)	42.72 (35.69)	**0.010**
Dyspnea	20.63 (24.67)	36.94 (28.09)	**0.043**
Insomnia	22.22 (26.53)	38.74 (27.79)	**0.043**
Appetite loss	12.70 (24.67)	26.13 (34.37)	0.190
Constipation	4.76 (11.95)	13.51 (24.16)	0.356
Financial disturbances	30.16 (31.46)	46.85 (39.64)	0.139

**Table 5:** Mean scores in The Short Form (36) Health Survey (SF-36) questionnaire subscales according to chronic graft-vs-host disease (cGVHD) activity and intensity of immunosuppression

SF-36 mean (standard deviation)	Patients with inactive cGVHD (n = 18)	Patients with active or highly active cGVHD (n = 20)	*P* (Mann-Whitney test)	Intensity of immunosuppression			*P* (Kruskal-Wallis test)
				no (n = 20)	moderate (n = 11)	high (n = 7)	
Physical functioning	70.40 (21.25)	41.0 (23.65)	**0.007**	62.25 (24.62)	59.36 (21.69)	30.71 (27.14)	**0.044**
Role limitations due to physical health	52.78 (41.91)	21.25 (36.52)	**0.026**	51.19 (42.19)	29.55 (40.02)	3.57 (9.45)	**0.031**
Role limitations due to emotional problems	59.25 (43.62)	36.66 (44.46)	0.161	49.20 (46.69)	63.64 (43.35)	23.80 (37.09)	0.195
Energy/fatigue	59.41 (18.53)	42.90 (18.20)	**0.017**	54.0 (22.98)	47.27 (15.55)	44.0 (14.73)	0.349
Emotional well-being	73.86 (13.71)	61.00 (19.23)	0.411	62.20 (22.04)	63.64 (16.92)	55.43 (13.75)	0.637
Social functioning	69.44 (21.53)	47.50 (34.28)	**0.014**	62.50 (25.92)	59.09 (22.42)	41.07 (31.22)	0.266
Pain	63.19 (19.68)	57.25 (34.28)	0.650	60.95 (25.76)	70.22 (28.01)	37.50 (26.38)	0.059
General health	49.04 (20.14)	39.44 (18.63)	0.279	48.69 (23.34)	40.34 (11.01)	35.0 (18.71)	0.428

**Table 6:** Mean scores in The EORTC Quality of Life Questionnaire C30 (QLQ C30) questionnaire subscales according to chronic graft-vs-host disease (cGVHD) activity and intensity of immunosuppression

QLQ C30 mean (standard deviation)	Patients with inactive cGVHD (n = 18)	Patients with active or highly active cGVHD (n = 20)	*P* (Mann-Whitney test)	Intensity of immunosuppression			*P* (Kruskal- Wallis test)
				no (n = 20)	moderate (n = 11)	high (n = 7)	
Global health status	63.42 (22.89)	44.58 (24.52)	**0.015**	59.12 (26.99)	55.30 (20.16)	33.33 (15.96)	**0.039**
Functional scales							
Physical functioning	75.0 (20.90)	56.50 (23.73)	**0.011**	72.06 (21.89)	63.03 (17.98)	49.52 (31.24)	0.096
Role functioning	68.52 (32.28)	44.17 (32.57)	**0.029**	61.91 (33.81)	59.09 (33.64)	33.33 (28.87)	0.142
Emotional functioning	66.20 (28.51)	60.0 (22.06)	0.427	64.29 (23.88)	62.12 (33.41)	55.95 (14.99)	0.583
Cognitive functioning	72.22 (24.15)	70.0 (27.36)	0.726	73.02 (26.60)	71.21 (31.70)	64.29 (26.22)	0.668
Social functioning	65.74 (24.41)	37.50 (31.93)	**0.009**	61.91 (31.69)	45.46 (28.96)	28.57 (34.31)	0.070
Symptom scale							
Fatigue	33.26 (20.16)	49.92 (29.59)	0.057	40.65 (23.39)	36.30 (27.69)	58.67 (31.18)	0.169
Nausea and vomiting	3.70 (7.13)	15.83 (24.47)	0.121	9.52 (22.71)	6.06 (11.24)	19.05 (14.99)	0.152
Pain	35.19 (31.77)	47.50 (39.09)	0.349	38.09 (35.80)	30.30 (33.18)	73.81 (21.21)	**0.033**
Dyspnea	31.48 (24.18)	40.0 (31.72)	0474	39.68 (29.09)	27.27 (25.03)	42.86 (31.71)	0.492
Insomnia	38.89 (26.19)	40.0 (29.81)	0.953	42.86 (30.08)	27.27 (20.10)	52.38 (26.23)	0.133
Appetite loss	16.67 (30.78)	33.33 (35.87)	0.147	25.39 (36.37)	21.21 (26.97)	33.33 (38.49)	0.814
Constipation	18.52 (28.52)	8.33 (18.33)	0.396	14.29 (24.88)	9.09 (21.56)	14.29 (9.91)	0.882
Diarrhea	5.56 (12.78)	10.53 (24.97)	0.785	7.94 (14.55)	3.03 (10.05)	16.67 (40.83)	0.805
Financial disturbances	46.29 (41.44)	46.67 (38.08)	0.971	34.92 (37.23)	57.58 (36.79)	57.14 (46.0)	0.234

## Discussion

In this study we investigated the ability of standard QOL questionnaires, SF-36 and QLQ C30, to evaluate QOL in patients with cGVHD graded according to NIH consensus criteria. Previous reports have identified cGVHD as the most important predictor of adverse long-term late effects and poor overall health after allo-SCT (3,35,36). Moreover, previous studies have mostly shown the adverse impact of cGVHD on health-related QOL (15-20). The limitations in assessing the results of these reports are related to their heterogeneous nature. Also, most of these studies evaluated QOL with regard to cGVHD without reviewing its severity or by grading the severity according to the old scoring system developed in the 1980s (37). Only one study examined the relationship between cGVHD severity defined by the new NIH Consensus criteria and QOL, and this was done with two standard QOL questionnaires – SF-36 and Functional Assessment of Cancer Therapy-Bone Marrow Transplant (FACT-BMT). This study found cGVHD severity to be independently associated with QOL in both questionnaires, adjusted for age (38).

In our study, older age, female sex, and short follow-up were not found to be predictive factors of worse QOL, as it was shown before (18,39,40). However, we confirmed that patients with cGVHD reported significantly lower QOL than control patients without cGVHD on all functional scales and general well-being evaluated by SF-36. Moreover, QOL of patients without cGVHD was comparable to normative data of the Croatian population measured with SF-36 (30). To the best of our knowledge, this is the first report to evaluate QOL with EORTC QLQ C30 questionnaire in patients with cGVHD defined by the NIH Consensus criteria. Compared with previous findings on QLQ C30 in allo-SCT, which show improvement of QOL scores over time (41), our patients without cGVHD had similar mean QOL scores as patients more than 3 years after allo-SCT, while cGVHD patients had scores comparable to QOL of patients at discharge from the hospital after allo-SCT. In our study population the differences between cGVHD patients and no-cGVHD controls were most evident in global health status, and physical and social well-being. These results indicate that C30 adds more information on social well-being of cGVHD patients and should be used as a valuable instrument of evaluation in the social domains of QOL.

When we compared patients with moderate cGVHD to patients with severe cGVHD, patients with moderate cGVHD had higher QOL subscale scores, but the difference was not significant, presumably due to the small number of patients. For the same reason, we could not compare the difference in QOL between patients with mild and moderate cGVHD, as we had only one patient with mild cGVHD. This is probably due to the referral pattern of these patients to our institution and some of the mild cGVHD have probably stayed undetected. Additionally, we evaluated QOL in cGVHD patients according to the cGVHD activity and intensity of immunosuppressive treatment. As patients with inactive cGVHD and patients without immunosuppressive therapy had better QOL scores, our results confirmed that the resolution of cGVHD and discontinuation of immunosuppressive treatment reduced QOL impairment in cGVHD patients.

Our study has a number of limitations. We used a group of patients from a single institution, and patients were included in the study at different time points after allo-SCT using heterogeneous transplant regimens. However, the patients were also part of a prospective observational study and therefore they had to meet the same inclusion criteria and were evaluated in a homogenous manner. Nevertheless, our ability to draw conclusions in a heterogeneous patient group supports the use of both SF-36 and C30 as valuable tools in the evaluation of the effect of GVHD on QOL in different clinical settings.

Today, there is a widespread interest in possible interventions to improve QOL in long-term allo-SCT survivors. Behavioral interventions, such as supervised exercise during hospitalization for allo-SCT, have been shown to result in better long-term physical well-being, decreased fatigue, and emotional distraction (42-44). Furthermore, psychosocial interventions, such as stress management and coping skills training reduced pain in allo-SCT survivors (45,46). Finally, a dedicated GVHD clinic with an active involvement of specialists interested in GVHD can also improve QOL of patients with cGVHD (47).

In conclusion, we confirmed QOL as a valid measure of cGVHD defined and staged by NIH consensus criteria. The results suggest that SF-36 is a useful questionnaire in the evaluation of cGVHD effect on QOL, particularly in the physical domains, while QLQ C30 provides additional value, especially in social domains. Future studies are needed to continue longitudinal QOL assessment by the same means in order to improve the outcomes of cGVHD patients.
